# Polo-like kinase 4 mediates epithelial–mesenchymal transition in neuroblastoma via PI3K/Akt signaling pathway

**DOI:** 10.1038/s41419-017-0088-2

**Published:** 2018-01-19

**Authors:** Xiangdong Tian, Dejun Zhou, Lu Chen, Yao Tian, Benfu Zhong, Yanna Cao, Qiuping Dong, Meng Zhou, Jie Yan, Yalei Wang, Yanli Qiu, Lianmin Zhang, Zhongyuan Li, Huijuan Wang, Daowei Wang, Guoguang Ying, Qiang Zhao

**Affiliations:** Tianjin Medical University Cancer Institute and Hospital, National Clinical Research Center for Cancer, Key Laboratory of Cancer Prevention and Therapy of Tianjin, Tianjin’s Clinical Research Center for Cancer, Tianjin, People’s Republic of China

## Abstract

Neuroblastoma (NB) is the most common malignant tumor in infancy and most common extracranial solid tumor in childhood. With the improvement of diagnosis and treatment, the survival rate of patients with low-risk and intermediate-risk NB can reach up to 90%. In contrast, for high-risk NBs, the long-term survival rate is still <40% because of heterogeneity of this tumor. The pathogenesis of NB is still not explicit, therefore it is of great significance to explore the mechanism of NB tumorigenesis and discover new therapeutic targets for NB. Polo-like kinase 4 (PLK4), one of the polo-like kinase family members, is an important regulator of centriole replication. The aberrant expression of PLK4 was found in several cancers and a recent study has unraveled a novel function of PLK4 as a mediator of invasion and metastasis in Hela and U2OS cells. However, the function of PLK4 in NB development and progression remains to be elucidated. The study showed the expression level of PLK4 in NB tissues was remarkably upregulated and high expression of PLK4 was negatively correlated with clinical features and survival, which suggested that PLK4 could be a potential tumor-promoting factor of NB. Functional studies indicated downregulation of PLK4 suppressed migration and invasion and promoted apoptosis in NB cells. Further experiments showed that downregulation of PLK4 in NB cells inhibited EMT through the PI3K/Akt signaling pathway. Animal experiments demonstrated that the downregulation of PLK4 in SK-N-BE(2) cells dramatically suppressed tumorigenesis and metastasis. PLK4 may be a promising therapeutic target for NB.

## Introduction

Despite improvement in diagnosis and therapy, neuroblastoma (NB) remains a vital health problem for children, which is the most commonly occurring extracranial solid tumor in childhood^1^. It accounts for 7–10% of all pediatric malignancies but is disproportionately responsible for the percentage of children’s cancer deaths, nearly 15%^2–4^. Systemic multidisciplinary therapy has increased the overall survival (OS) rate up to 90% or so for children with NB.^4,5^ But the extraordinary degree of heterogeneity in NB makes it vary from spontaneous regression to a very aggressive form^6,7^. Despite intensive multimodality therapy, unfortunately, relapse and metastasis remain common among patients with high-risk NB and the long-term survival rate is <40%^8,9^. Recent progress in the treatment of this group of patients may be owing to the use of immunotherapy such as GD2 antibody ch14.18 and other novel targeted agents^10–13^. Therefore, there is an urgent need to reveal the mechanisms underlying NB tumorigenesis and metastasis and to explore promising new therapies for the tumor.

In spite of different characteristics and pathogenesis in diverse cancers, an unprecedented proliferation rate is a common feature of all tumor cells^14,15^. Centrosome amplification is often observed in human tumors, which is speculated to induce centrosome instability and tumorigenesis^16,17^. Polo-like kinase 4 (PLK4), also known as Sak, maps to a chromosome region, 4q28, and localizes to centrosomes with the function of regulation of centrosome duplication^18–20^. Recently, it has been demonstrated that PLK4 enhances cancer cell invasion and Hela cells could be regulated from a classic mesenchymal to a more epithelial phenotype by down-regulating PLK4 expression^21^. Previous studies indicated that PLK4 was aberrantly expressed in several tumors of breast, liver, colon, and prostate, although its expression level differed among those cancers^22–24^. However, the function of PLK4 in NB tumorigenesis and metastasis remains to be elucidated. The transformation of a cell with epithelial characteristics into one with mesenchymal traits occurs during the epithelial–mesenchymal transition (EMT) process, which has been uncovered to play a significant role in tumor development^25^, and the activation of EMT is commonly reflected a vested feature of malignancy^26,27^. EMT was also reported to be associated with the migratory and invasive properties of human NB cells^28^. Phosphatidylinositol-3-kinase (PI3K) is a key signaling molecule in many cell activities, which can regulates cell division, differentiation, apoptosis and so on. Especially, there is increasing evidence that the PI3K/Akt pathway plays an important role in the development and progression of NB^29–31^. David King et al. even considered it as a novel therapeutic strategy in NB^32^. But the underlying mechanism is not fully understood. As the most thoroughly studied member of PLK family, PLK1 was reported to accelerate EMT in gastric and pancreatic cancer cells through the PI3K/Akt pathway^33,34^, whereas the relationship between PLK4 and PI3K/Akt pathway remains unknown.

In this study, we demonstrated that PLK4 expression positively correlated with primary site, serum level of lactate dehydrogenaserelapse (LDH), recurrence, expression level of Ki-67, clinical stage by International Neuroblastoma Staging System (INSS), and poor prognosis. Furthermore, we demonstrated that PLK4 induced NB cells to undergo EMT via the PI3K/Akt signaling pathway. Our results unraveled a novel function of PLK4 as a mediator of EMT in NB cell lines, implicating it as a potential target for the treatment of NB.

## Results

### PLK4 was elevated in NB tissues and high expression of PLK4 was a poor prognostic factor

**Fig. 1:**
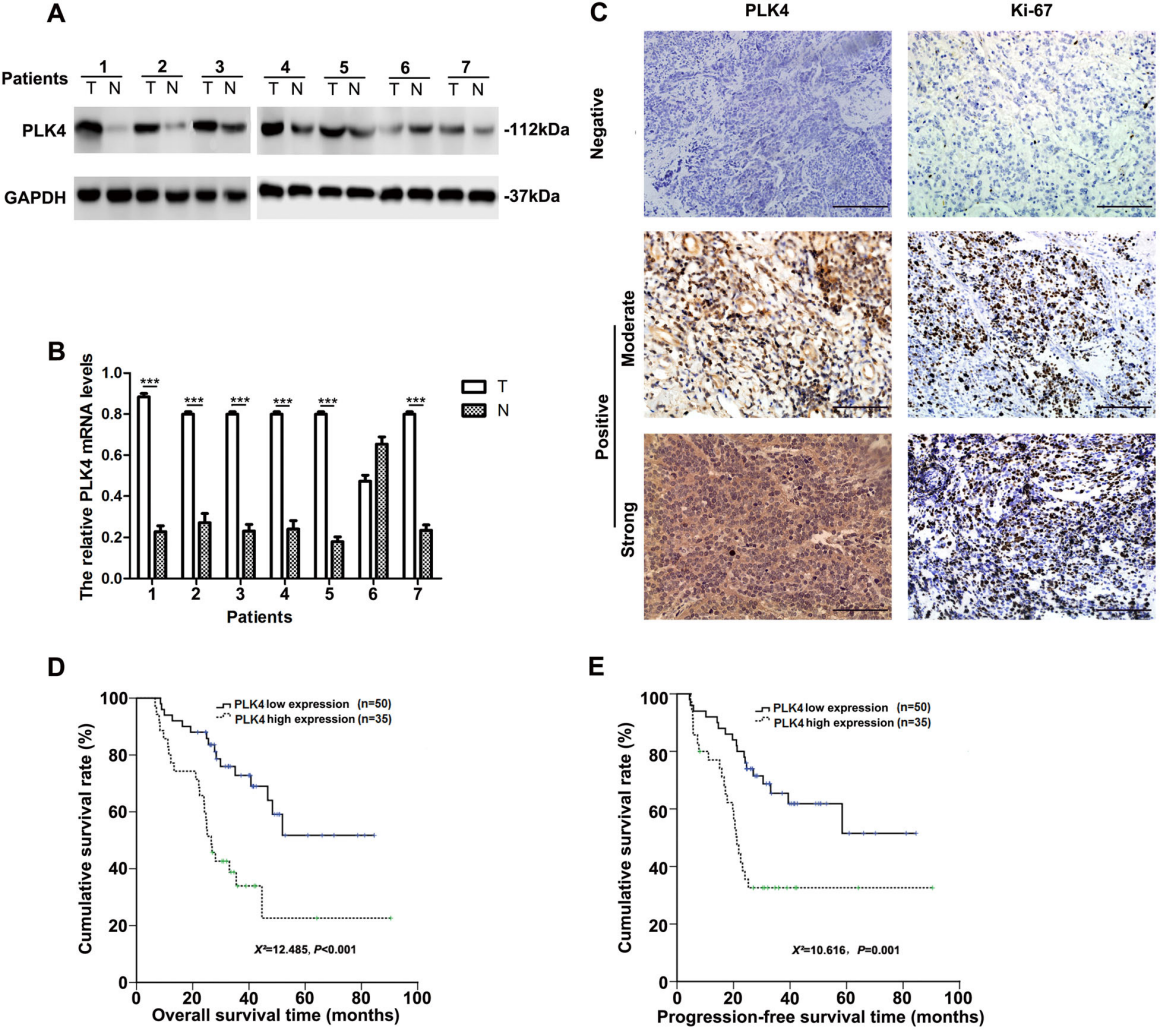
PLK4 is elevated in NB tissues and PLK4 high expression is a poor prognostic factor **a** Western blot analysis of PLK4 protein expression in NB tissues and paired normal tissues (T: tumor tissue, N: non-tumor tissue). **b** Real-time PCR analysis of PLK4 mRNA expression in NB tissues and relevant normal tissues. **c** Immunohistochemistry estimation of PLK4 and Ki-67 in NB tissues with different histological grades (scale bar, 1.0 mm). **d** Kaplan–Meier overall survival analysis of 85 NB patients with different PLK4 expression levels. **e** Kaplan–Meier progression-free survival analysis of 85 NB patients with different PLK4 expression levels. (***P < 0.001)

We initially randomly collected seven pairs of freshly and untreated NB and adjacent normal tissues to analyze the protein expression level of PLK4 by western blot (WB), the results showed that when compared with adjacent normal tissues, PLK4 was elevated in 6/7 collected NB tissues (Fig. 1a). Furthermore, real-time PCR (RT-PCR) indicated the similar results (Fig. 1b). Additionally, we evaluated the PLK4 expression levels in NB cell lines by WB. The results showed that the PLK4 expression was elevated in SK-N-SH and SK-N-BE(2) cell lines as compared with Hela and Du145 cell lines, which were known to be positive (Supplementary Figure 1A). Taken together, these findings indicated that PLK4 was elevated in NB. Immunohistochemistry (IHC) was applied to investigate the expression levels of PLK4 and Ki-67 in 85 patients enrolled in our hospital from 2009 to 2014 (Fig. 1c). We analyzed the relationships between PLK4 and clinicopathological variables, including sex, age, primary site, N-myc status, serum level of LDH, bone marrow metastasis at time of diagnosis, recurrence and expression level of Ki-67, clinical stage by INSS. The results were listed in Table 1. The expression level of PLK4 was closely related with primary site (*χ*^2^ = 4.093, *P* = 0.038), serum level of LDH (*χ*^2^ = 29.181, *P* < 0.001), recurrence (*χ*^2^ = 7.280, *P* = 0.006), expression level of Ki-67 (*χ*^2^ = 5.835, *P* = 0.016) and clinical stage by INSS (*χ*^2^ = 5.270, *P* = 0.019). However, there was no significant correlation between PLK4 expression and age, gender, or bone marrow metastasis (*P* > 0.05). The results suggested that PLK4 expression correlated with adverse clinical features in NB. We next assessed how PLK4 affected clinical outcomes by Kaplan–Meier survival analysis. It turned out that patients with high expression level of PLK4 had significantly worse 3-year OS (*χ*^2^ = 12.485, *P* < 0.001; Fig. 1d) and 3-year progression-free survival (PFS, *χ*^2^ = 10.616, *P* = 0.001; Fig. 1e) than those with low expression level of PLK4.

**Table 1:** The relationship between PLK4 expression and clinical pathological characteristics in 85 NB patients

Characteristics	Cases, *n*	PLK4		χ^2^	*P-*value
		Lower, *n*	Higher, *n*		
Sex					
Male	52	31	21	0.035	0.515
Female	33	19	14		
Age					
<18 months	14	10	4^a^	1.099	0.228
≥18 months	71	40	31		
Primary site					
Abdomen, cervix	69	37	32	4.093	0.038*
Pelvis, thorax	16	13	3^a^		
N-myc status					
Amplification	25	16	9	0.037	0.528
Unamplification	47	29	18		
Serum LDH level					
<1500 U/L	59	46	13	29.181	<0.001***
≥1500 U/L	26	4 ^a^	22		
Bone marrow metastasis					
Positive	62	33	29	2.964	0.069
Negative	23	17	6		
Recurrence					
Yes	41	18	23	7.280	0.006**
No	44	32	12		
Expression level of Ki-67					
Low	40	29	11	5.835	0.016*
High	45	21	24		
Clinical stage of INSS					
1, 2, 4s	11	10	1^a^	5.270	0.019
3, 4	74	40	34		

*LDH* lactate dehydrogenase, *INSS* International Neuroblastoma Staging System

*P   < 0.05, **P  <  0.01, ***P < 0.001

^a^Fisher test was used when *n* < 5

### PLK4 regulated NB cell apoptosis, migration, and invasion capacities in vitro

**Fig. 2:**
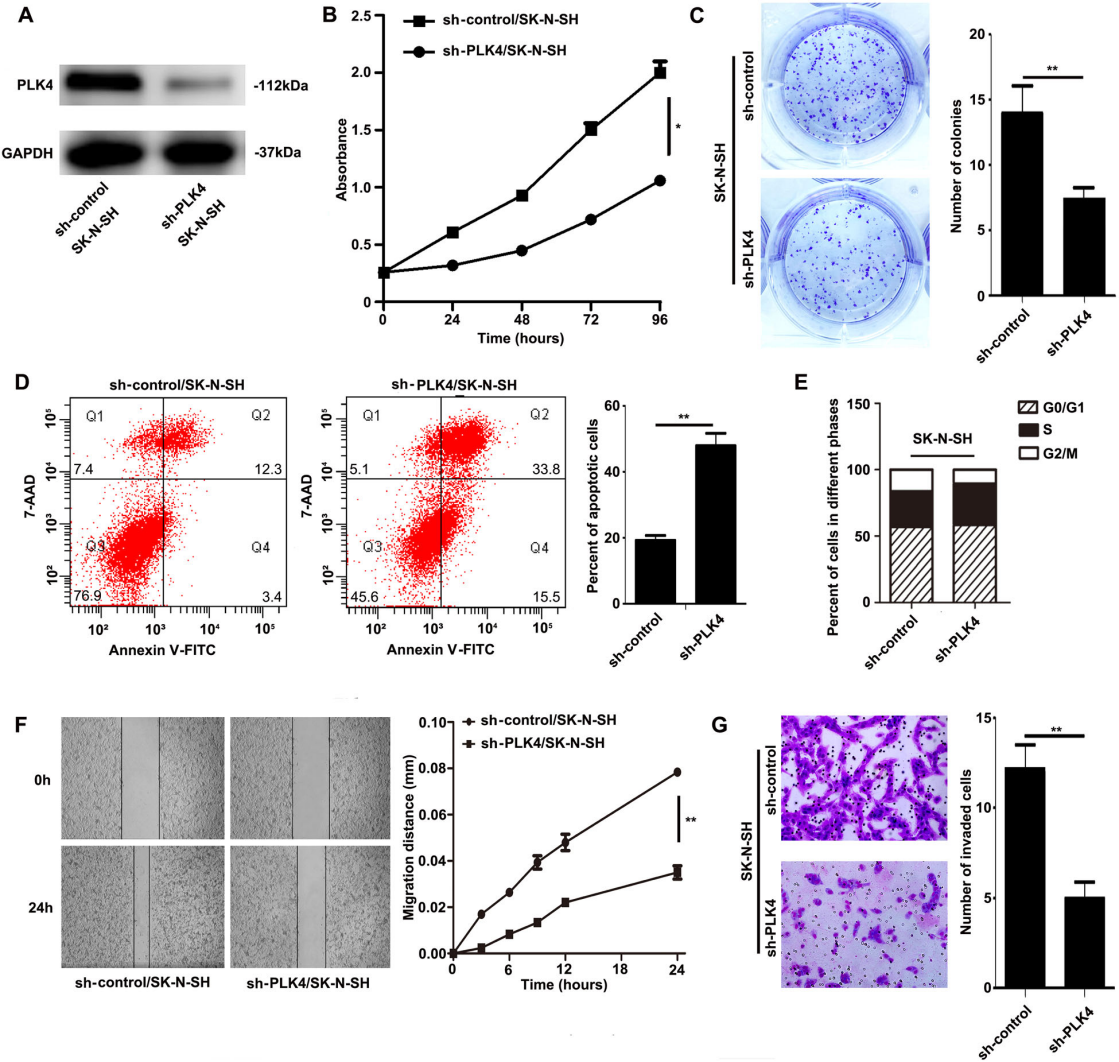
PLK4 regulated SK-N-SH cell apoptosis, migration, and invasion capacities *in vitro* **a** Assessment of the transfected efficiency of PLK4 protein expression after infection in SK-N-SH cells. Cell proliferation comparison by MTT assay (**b**, *P* = 0.0429) and colony formation assay (**c**, *P* = 0.0097) in sh-control and sh-PLK4 SK-N-SH cells. **d** SK-N-SH cells were stained with FITC Annexin V and 7-AAD, then percentage of apoptotic cells analyzed by flow cytometry in sh-control and sh-PLK4 SK-N-SH cells (*P* = 0.0025), Q4 and Q2 regions represent the rate of early and late apoptotic cells, respectively. **e** Cell cycle analysis by flow cytometry in sh-control and sh-PLK4 SK-N-SH cells and the percentage of cells in the G0/G1, S, or G2/M phases of the cell cycle is indicated. **f** Scratch assay comparing the migration in sh-control and sh-PLK4 SK-N-SH cells (*P* = 0.0086). **g** Comparison of the invasion potential in sh-control and sh-PLK4 SK-N-SH cells by counting the number of the cells that invaded through matrigel-coated transwell inserts (*P* = 0.005). All experiments were repeated at least three times. (*P < 0.05, **P < 0.01)

**Fig. 3:**
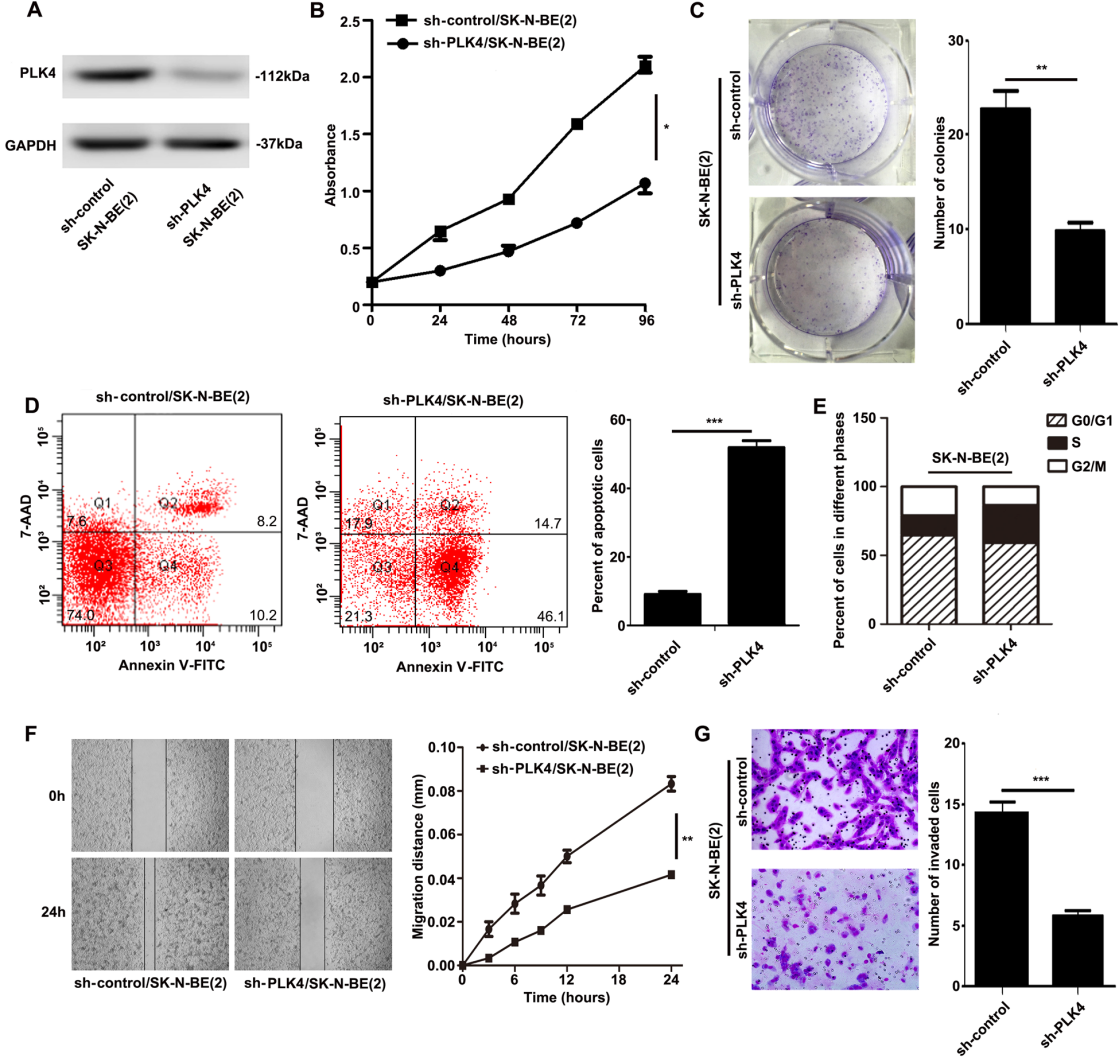
PLK4 affected SK-N-BE(2) cell biological characteristics **a** Assessment of the transfected efficiency of PLK4 protein expression after infection in SK-N-BE(2) cells. Cell proliferation comparison by MTT assay (**b**, *P* = 0.0475) and colony formation assay (**c**, *P* = 0.0065) in sh-control and sh-PLK4 SK-N-BE(2) cells. **d** SK-N-BE(2) cells were stained with FITC Annexin V and 7-AAD, then percentage of apoptotic cells analyzed by flow cytometry in sh-control and sh-PLK4 SK-N-BE(2) cells (*P* < 0.001), Q4 and Q2 regions represent the rate of early and late apoptotic cells, respectively. **e** Cell cycle analysis by flow cytometry in sh-control and sh-PLK4 SK-N-SH cells and the percentage of cells in the G0/G1, S, or G2/M phases of the cell cycle is indicated. **f** Scratch assay comparing the migration in sh-control and sh-PLK4 SK-N-BE(2) cells (*P* = 0.0093). **g** Comparison of the invasion potential of sh-control and sh-PLK4 SK-N-BE(2) cells by counting the number of the cells that invaded through matrigel-coated transwell inserts (*P* < 0.001). All experiments were repeated at least three times. (*P < 0.05, **P < 0.01, ***P < 0.001)

According to the above findings, we considered that PLK4 was a biologic reason for aggression of NB. First, the downregulation effect of PLK4 in ΝΒ cells was verified by WB (Figs. 2a, 3a). Then, cell proliferation, migration, and invasion abilities were compared between sh-control and sh-PLK4 NB cells in vitro. Downregulation of PLK4 resulted in a lower proliferation rate in SK-N-SH and SK-N-BE(2) cells as assessed by MTT and colony formation assays. The MTT assay showed that downregulation of PLK4 resulted in a substantial decrease in the rate of cell proliferation (Figs. 2b, 3b). By colony formation assay, we found that the number of colonies in the group of cells with decreased PLK4 was evidently reduced (Figs. 2c, 3c).

To confirm whether proliferation rate was affected by downregulation of PLK4, we further detected the cell cycle and apoptosis in the two groups of NB cells. Consequently, compared with sh-control NB cells, the percentage of apoptotic cells was found to be significantly higher in sh-PLK4 NB cells (Figs. 2d, 3d). As shown in Figs. 2e and 3e, compared with sh-control NB cells, there existed an increase in the S phase and a reduction in the G2/M phase in sh-PLK4 NB cells, without changes in G0/G1 phase. Taken together, the above results indicated that downregulation of PLK4 suppressed the proliferation rate through prompt of apoptosis and delay in the S phase in NB cells.

Furthermore, we investigated the role of PLK4 in NB cell migration and invasion through scratch assay and matrigel invasion assays. In the scratch assay, the relative migration distance of sh-PLK4 NB cells was significantly shorter than that of sh-control NB cells (Figs. 2f, 3f). The analysis of cell invasion ability with matrigel invasion assay showed similar result. Sh-PLK4/SK-N-SH cells distinctly reduced cell migration and invasion (Fig. 2g). Similar experiments were performed in SK-N-BE(2) cells (Fig. 3g), which demonstrated the same result.

### PLK4 promoted EMT in NB

**Fig. 4:**
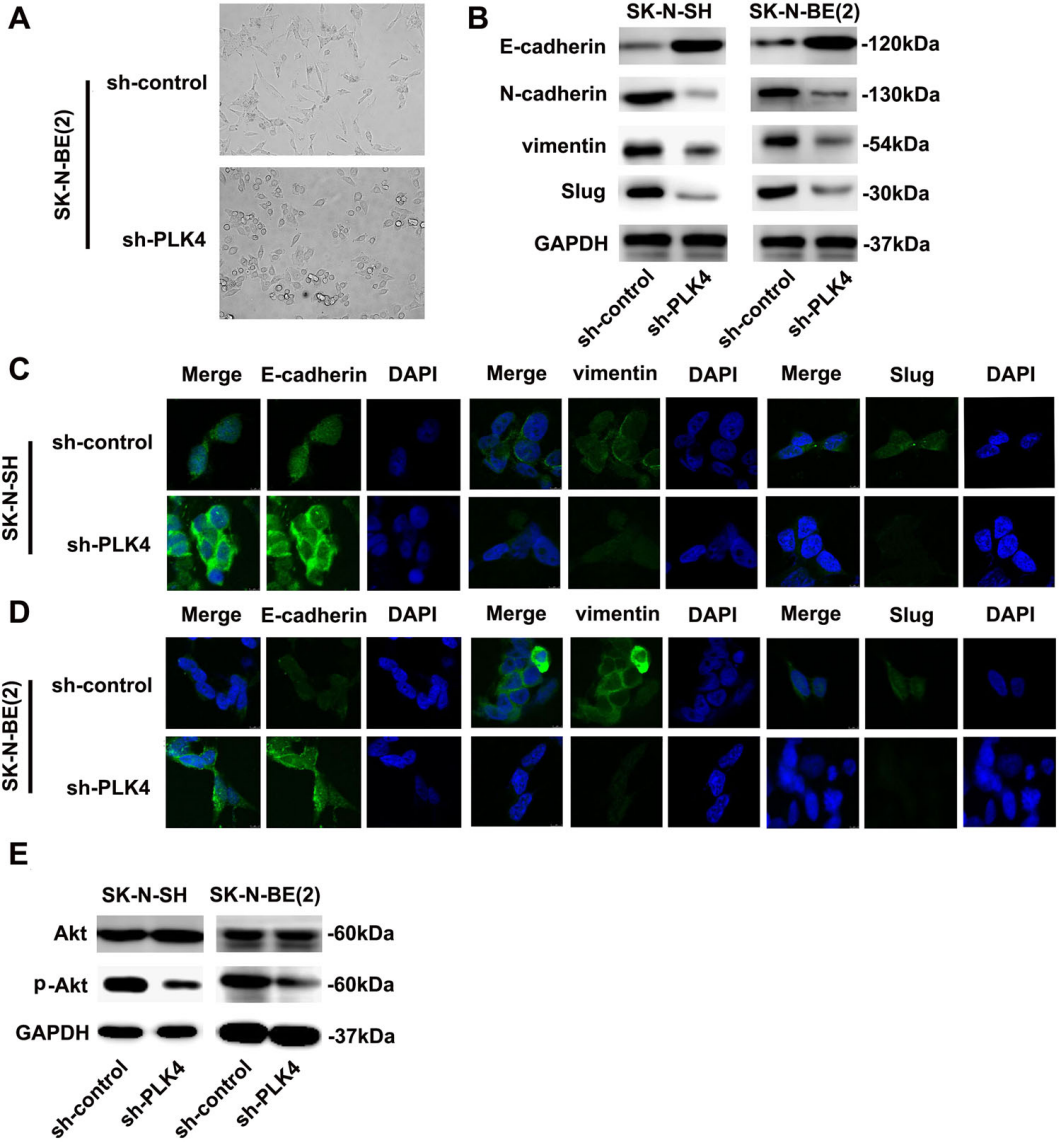
PLK4 promoted epithelial–mesenchymal transition (EMT) in NB cells **a** Morphologic change in SK-N-BE(2) cells when PLK4 was downregulated. **b** Western blot of EMT-associated markers expression (epithelial marker E-cadherin, mesenchymal markers N-cadherin, vimentin, and Slug) in NB cell lines when PLK4 was downregulated. Immunofluorescence staining for EMT-associated markers (epithelial marker E-cadherin, mesenchymal markers vimentin, Slug) of sh-control and sh-PLK4 in SK-N-SH **c** and SK-N-BE(2) **d** cells, respectively. **e** Western blot of total Akt and phosphorylated Akt in sh-control and sh-PLK4 NB cells

When PLK4 was downregulated in SK-N-BE(2) cells, we noticed that cells turned from fibroblast-like morphology to cobblestone-like morphology compared with sh-control/SK-N-BE(2) cells (Fig. 4a). According to the above phenomenon, we speculated that PLK4 might play a role in EMT of NB cells, which has been verified to promote cancer cell migration and invasion^35,36.^ To examine the influence of PLK4 expression on EMT in NB, we measured the expression of epithelial and mesenchymal markers by WB and IF. The results indicated that the expression level of epithelial marker E-cadherin increased, whereas the mesenchymal markers such as N-cadherin, vimentin and Slug decreased in sh-PLK4 NB cells (Fig. 4b). Similar results were obtained by IF (Figs. 4c, d, Supplementary Figure 1D). Considering the findings mentioned above, we drew a conclusion that PLK4 regulated EMT in NB cells through EMT-associated transcription factors.

EMT is regulated through several signaling pathways, including Akt, Notch, and NF-κB^37–39^. Additionally, Akt can directly affect the morphology, migration, invasion, and tumorigenesis of tumor cells. Activated PI3K/Akt signaling pathway was identified to play a significant role in regulation of EMT through mediation of several transcriptional and growth factors^40–43^. And PI3K/Akt pathway was reported to participate in the development and progression of NB^29–31^. As was shown in Fig. 4e, the phosphorylation of Akt remarkably decreased in sh-PLK4 NB cells, whereas it had no impact on total Akt with WB. We hypothesized that PLK4 regulated the EMT process through the PI3K/Akt signaling pathway. To confirm this assumption, we cultured pCDH-PLK4 NB cells with an exogenous specific inhibitor of PI3Kinase pathway, LY294002. We observed that LY294002 inhibited the upregulation of p-Akt in pCDH-PLK4 NB cells (Supplementary Figure 2B). Moreover, in the group of pCDH-PLK4 NB cells interfered with the inhibitor, expression of epithelial biomarker E-cadherin was elevated, whereas the mesenchymal biomarkers (N-cadherin, vimentin, and Slug) were downregulated confirmed by WB (Supplementary Figure 2C).

**Fig. 5:**
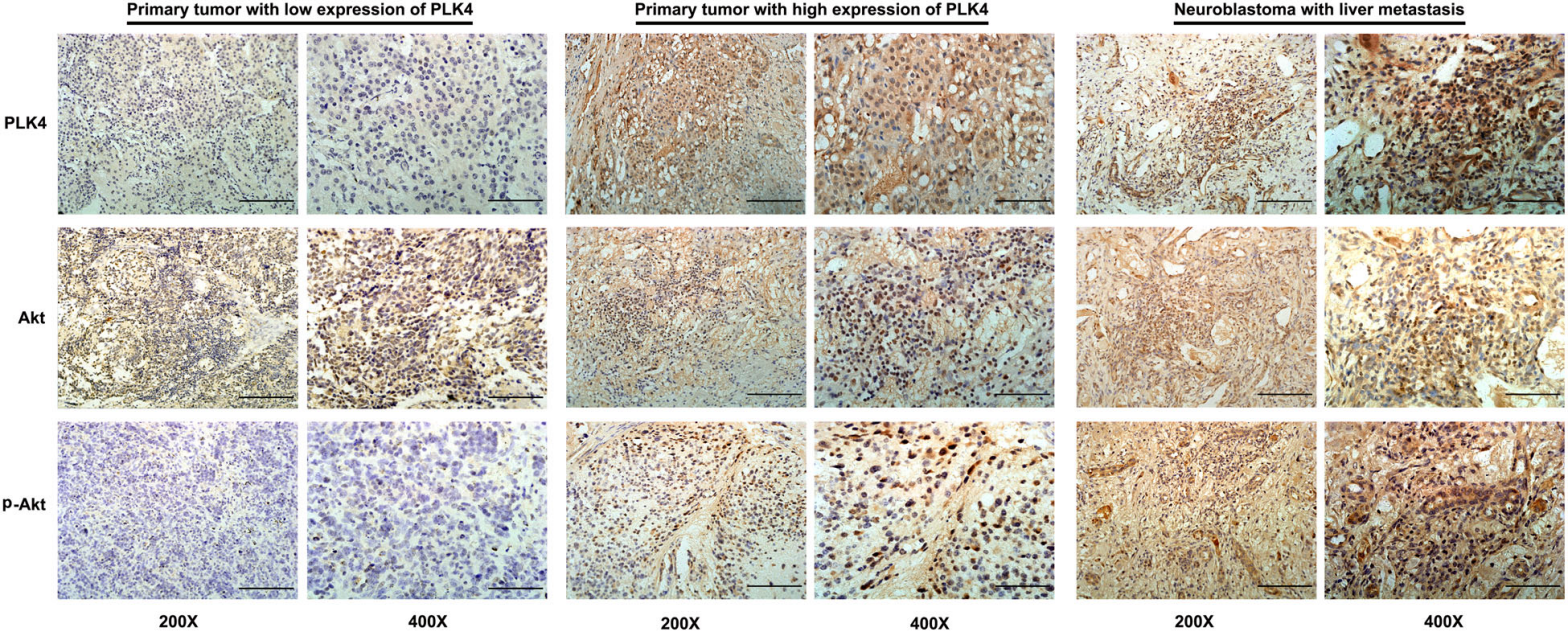
Comparison of expression levels of Akt and p-Akt in NB tissues No different expression level of Akt among tissues with low expression of PLK4, high expression of PLK4, and liver metastasis. The expression levels of PLK4 and p-Akt in neuroblastoma liver metastasis were higher than those in primary tumor. (Scale bar, 1.0 mm)

To further confirm that PLK4 regulated the EMT process via the PI3K/Akt signaling pathway, we examined the expression level of Akt and p-Akt in NB tissues with IHC. More than half of children diagnosed with NB present metastatic disease^1^. And the most frequently metastatic site is bone marrow while the presence of metastasis in solid organ is relatively rare^44,45^. Two patients with liver metastasis were selected to evaluate the expression level of PLK4. Higher expression level of PLK4 was found in NB liver metastasis tissues, rather than primary tumor tissues. Then, expression levels of Akt and p-Akt were assessed in NB tissues. The results showed that the expression level of Akt was stable among primary tumor tissues with diverse expression level of PLK4, whereas expression level of p-Akt in tissues of NB liver metastasis was higher than that of primary tumors (Fig. 5).

In sum, these results suggested that PLK4 promoted NB progression through activation of PI3K/Akt signaling pathway.

### Downregulation of PLK4 in SK-N-BE(2) cells suppressed tumorigenesis and inhibited metastasis in vivo

**Fig. 6:**
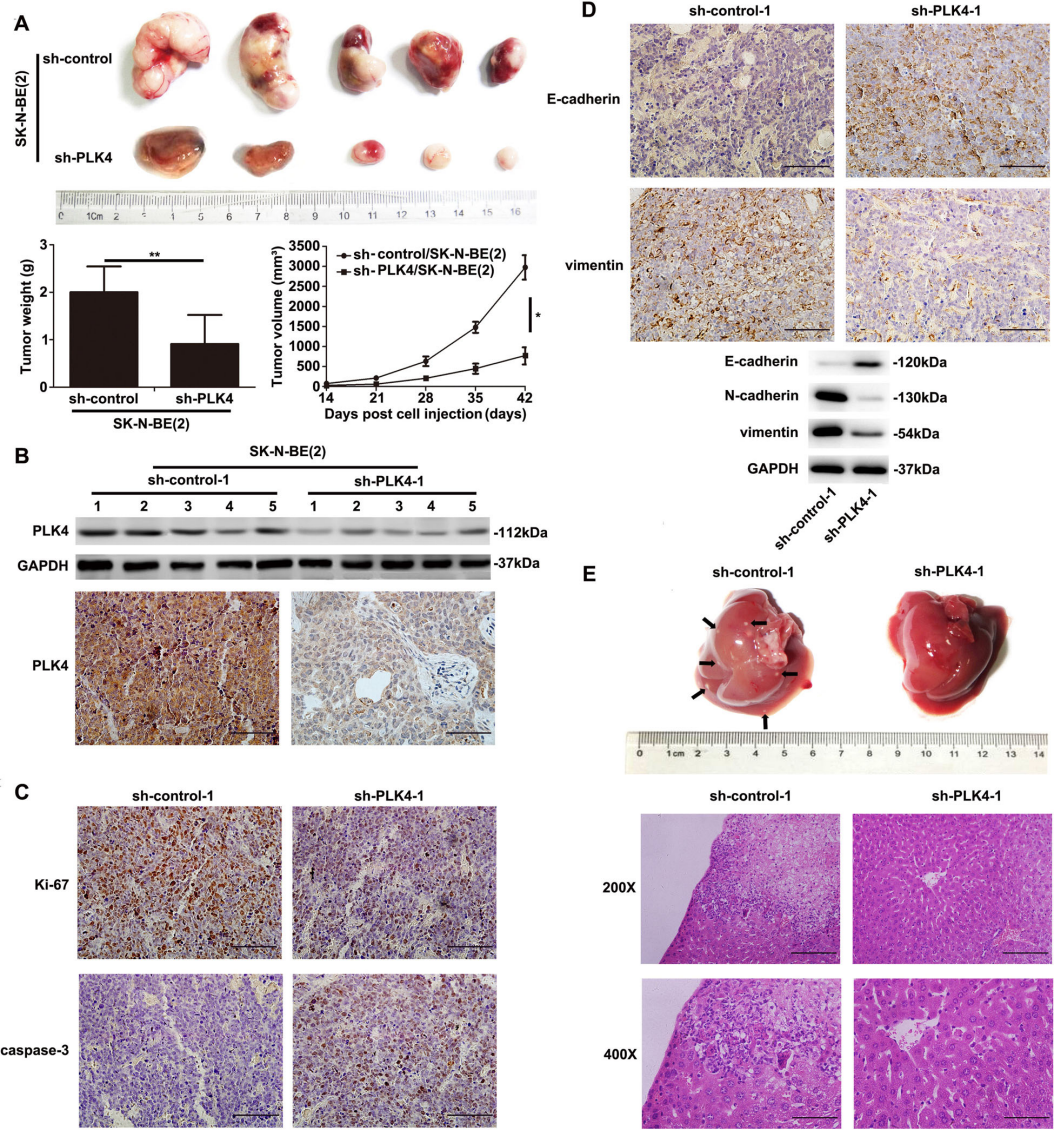
Downregulation of PLK4 in SK-N-BE(2) cells suppressed tumorigenesis and inhibited metastasis in vivo **a** Tumors from the nude mice that were transplanted with the same number of sh-control and sh-PLK4 SK-N-BE(2) cells (2.5 × 10^6^ cells per mouse) into the inguen (*n* = 5 per group), 6 weeks later all the mice were sacrificed then evaluated the primary tumors weight (*P* = 0.0061) and volume (*P* = 0.041) for two groups. **b** PLK4 expression level estimated by western blot and immunohistochemistry in primary tumors of two groups. **c** Assessment of proliferation and apoptosis expression with immunohistochemistry in primary tumors of two groups. **d** Immunohistochemistry and western blot results of EMT-associated markers expression estimation in the first mouse primary tumor of two groups. **e** Confirmation of hepatic metastases by hematoxylin and eosin staining. (*P < 0.05, **P < 0.01) (Scale bar, 1.0 mm)

To explore the function of PLK4 in tumorigenesis and metastasis in vivo, sh-control/SK-N-BE(2) cells and sh-PLK4/SK-N-BE(2) cells were inoculated into the inguens of nude mice (2.5 × 10^6^ per mouse). Tumors in the group of sh-control/SK-N-BE(2) cell injection could be touched a week later, while another week later, tumors in the sh-PLK4/SK-N-BE(2) cell-injection group could be palpable. Tumor weight and size were measured every 3 days. Six weeks after inoculation, one mouse became moribund and we euthanized all the mice. Then, mice tumor sizes, weights, and suspicious metastases in the two groups were assessed. Visually, tumor sizes in the sh-PLK4/SK-N-BE(2) cell injection group were smaller than those of sh-control/SK-N-BE(2) cell injection. And as drawn in the growth curve, it illustrated that PLK4 promoted tumorigenesis through increasing tumor weight and volume in vivo (Fig. 6a), which was consistent with the previous consequences in vitro. WB analysis and IHC were used to evaluate the expression level of PLK4, and PLK4 expression decreased in the sh-PLK4/SK-N-BE(2) cell injection group than that in the sh-control/SK-N-BE(2) cell injection group (Fig. 6b).

To analyze if there exists impact of PLK4 on the proliferative and apoptotic rate in vivo, IHC was used to observe the expression level of proliferative and apoptotic biomarkers. It turned out that higher expression level of Ki-67 and lower expression level of caspase-3 were observed in the sh-control/SK-N-BE(2) cell injection group than those of sh-PLK4/SK-N-BE(2) cell injection group (Fig. 6c).

Next, we assessed the correlation between PLK4 and EMT-associated markers in primary tumors of the first mouse during the two different groups using WB analysis and IHC (Fig. 6d). The results were parallel with the previous experimental consequences in vitro. Mesenchymal markers, such as N-cadherin and vimentin, were downregulated in the tumor of sh-PLK4/SK-N-BE(2) cell injection group, whereas the epithelial marker E-cadherin was upregulated.

Suspicious lesions were found in the liver of the first mice from the sh-control/SK-N-BE(2) cell injection group, which were confirmed to be hepatic metastases by hematoxylin and eosin (HE) staining (Fig. 6e). It might explain why the expression level of PLK4 is significantly correlated with the advanced stage of NB.

In brief, the results showed that downregulation of PLK4 in SK-N-BE(2) cells suppressed tumorigenesis and inhibited the metastasis of SK-N-BE(2) cells in vivo.

## Discussion

It has been reported that PLK4 is highly expressed in several tumor types, such as breast, colorectal cancers, glioblastoma, and bladder cancer^22,46^. Without doubt, owing to its significant effect on regulating the centrosome duplication^19,20^, abnormal mitosis, centrosomal amplification (CA), as well as chromosomal instability (CIN) is probably the most immediate outcome of aberrant PLK4^47,48^. And a causal connection between CA, CIN, aneuploidy, and tumorigenesis has been reported in previous studies^24,49^. The contribution of PLK4 to NB biology is also likely supported by its interaction with the Arp2/3 complex^21^, which further affects cell motility and polarity.

Elevated expression level of PLK4 mRNA is related with aggression and resistance to traditional therapy in breast cancer^46,50,51^. In parallel with previous studies, we found that the expression level of PLK4 was higher in NB tissues compared with adjacent normal tissues. In the present study, PLK4 expression was found to be closely correlated with risk factors and poor prognosis for NBs. Kazazian et al. indicated PLK4 enhanced migration and invasion in Hela and U2OS cells.^21^ Consistent with the study, we further confirmed PLK4 promoted migration and invasion in vitro and in vivo. Furthermore, downregulation of PLK4 facilitated NB cells apoptosis. In short, PLK4 facilitates aggressiveness of NB, which may explain the results that the expression level of PLK4 correlates with adverse clinical features and poor survival.

EMT is an important process in tumor progression and metastasis, and 90% of tumors show different degrees of EMT during tumor development^52–54^. During EMT, epithelial cells lose their characteristics, such as cell adhesion and polarity, reorganize their cytoskeleton, and gain mesenchymal phenotype properties including migration and invasion^36,55^. Recent studies indicated that EMT was a feature during the tumorigenesis, tumor progression, and drug resistance in NB^56–58^. Further researches have elucidated suppression of EMT and subsequent inhibition of cell proliferation and invasion^59^. Furthermore, EMT could be reversible even though mesenchymal to epithelial transition was often an incomplete process^60–62^. In our study, we found that downregulation of PLK4 depressed EMT program in NB cells, including upregulation of the epithelial marker E-cadherin, as well as downregulation of mesenchymal markers (N-cadherin, Slug, and vimentin). That is to say, there existed a shift from a classic mesenchymal to a more epithelial phenotype. Consistent with the previous study, Kazazian et al. found PLK4 depletion suppressed cancer invasion and regulated cancer cell shape in Hela and MDA-MB-231 cells.^21^ Although the process of mesenchymal to epithelial transition mediated by downregulation of PLK4 was incomplete, changes in cell migration and invasion did occur.

The PI3K/Akt signaling pathway can induce the EMT process and inhibit the transcription of E-cadherin^63^, which has been commonly considered as an activator of cancer progression. Akt could directly affect cell biological characteristics, which is shown to downregulate E-cadherin expression and promote EMT-like transition and invasiveness in carcinoma cells. The activation of PI3K/Akt pathway contributes to EMT through upregulation of EMT-related factors such as Snail, Slug, and so on^40,42,43,64^. Our study observed that the phosphorylation of Akt in pCDH-PLK4 NB cells was blocked by LY294002. And the expression level of p-Akt was significantly accompanied by PLK4 expression in NB tissues. These findings show that PLK4 mediates EMT in NB cells via influence on the PI3K/Akt signaling pathway, ultimately improving their migratory and invasive potential.

### Conclusions

In the present study, we demonstrate that PLK4 is upregulated in NB and PLK4 is significantly correlated with survival rate of NBs. Downregulation of PLK4 in NB cells facilitates cell apoptosis while suppressing cell migration and invasion. Furthermore, PLK4 induces EMT through the PI3K/Akt signaling pathway and may be a promising therapeutic target for NB.

## Materials and methods

### Clinical samples and cell culture

This study was approved by the Ethics Committee of Tianjin Medical University Cancer Institute and Hospital. All guardians have signed the informed consent. The clinical samples used in this study were all obtained from department of pediatric oncology. SK-N-SH, SK-N-BE(2), Hela, Du145, and HEK293T cell lines were purchased from the Type Culture Collection of the Chinese Academy of Sciences (Shanghai, China). SK-N-SH, SK-N-BE(2), Hela and four transfected cell lines, sh-control/SK-N-SH, sh-PLK4/SK-N-SH, sh-control/SK-N-BE(2), and sh-PLK4/SK-N-BE(2), were all cultured in minimum essential medium (MEM; Gibco, Carlsbad, CA, USA) supplemented with 10% fetal bovine serum (FBS, HyClone, USA). The Du145 and HEK293T cell lines was cultured in Dulbecco’s modified Eagle’s medium (Gibco) supplemented with 10% FBS (HyClone). All cells were supplemented with 1% penicillin–streptomycin solution (PS, HyClone) and cultured in a 5% CO_2_ and humidified incubator maintained at 37 °C.

### Plasmids and cell infection

The short hairpin RNAs (shRNAs) were cloned into a vector plsi-shRNA/copGFP. 5′-GCATCTCAAGAATATGTGAAATTTCACATATTCTTGAGATGC-3′ and 5′-GACCTTATTCACCAGTTACTTAAGTAACTGGTGAATAAGGTC-3′ were designed as PLK4 shRNA sequence respectively. PLK4 complementary DNA (cDNA) were subcloned into XbaI and BamHI sites of pCDH-CMV-MCS-EF1-puro lentiviral vector. The forward and reverse sequence were 5′-TCTAGAGCCACCATGGCGACCTGCATCGGGGAG-3′and 5′-GCTAGCTCAATGAAAATTAGGAGTCGG-3′, respectively. PLK4 shRNA plasmid and pCDH-CMV-MCS-EF1-puro were constructed in tumor cell biology laboratory of Tianjin Medical University Cancer Institute and Hospital. And the control shRNA sequence was synthesized by Shanghai Genechem Co., Ltd (Shanghai, China). HEK293T cells were used to conduct lentivirus. SK-N-SH and SK-N-BE(2) cells were prepared to be transfected. Thirty-five millimeter dishes placed with 2 × 10^5^ cells were infected by lentivirus for 6 h. Transfection efficiency with down-expression lentivirus was evaluated by using GFP (green fluorescent protein) as a report gene (Supplementary Figure 1B), plasmid with higher transfection efficiency was selected to make further experiments. Puromycin was used to screen cells that have been transformed by vectors with PLK4 overexpression. Then, the transfected cells were cultured for another 48 h and the expression level of PLK4 in cell lines were analyzed by WB technique.

### Immunohistochemistry assays

To avoid the interference of treatment in the analysis, tissues for the IHC were most obtained by needle biopsy or operation ahead of neoadjuvant chemotherapy. Paraffin-embedded tissue of NB were deparaffinized and rehydrated with xylene and graded concentrations of ethanol. In all, 3% H_2_O_2_ was used to block endogenous peroxidase activity for 15 min and nonspecific staining was blocked by 3% bovine serum albumin (BSA; Roche, HK, China) for 1 h. The incubation with the PLK4 (1:150) (Proteintech Group, Inc., Chicago, IL, USA), Ki-67 (1:200) (Zhongshan Goldbridge Biotechno-logy CO., Ltd, Beijing, China), Akt (Ser473, 1:100) (Cell Signaling Technology, Danvers, MA, USA), p-Akt (Ser473, 1:100), caspase-3 (1:50) (Santa Cruz Biotechnology, Inc., CA, USA), E-cadherin (1:100) (Sino Biological Inc., Beijing, China), or vimentin (1:1000) (Epitomics, CA, USA) antibodies occurred at 4 °C overnight. The next day, the tissues were placed at room temperature for half an hour and washed with phosphate-buffered saline (PBS) three times. Then, tissues were stained with the secondary antibody (Zhongshan Goldbridge Biotechno-logy CO., Ltd) at 37 °C for 1 h, washed with PBS three times, visualized by 3,3-diaminobenzidine staining and then counterstained with 10% Mayer hematoxylin, dehydrated, mounted, dried, and observed.

The staining intensity was classified on the scale of 0–3 (0 for no staining, 1, 2, 3 for weak, moderate and strong immunoreactivity, respectively). The percentage of positive cells was scored as 0 (0% positive cells), 1 (<30% positive cells), 2 (30–60% positive cells), and 3 (>60% positive cells). The scores of staining intensity and percentage of positive cells were multiplied as the final scores of positive staining. We finally defined two expression levels of the staining: sections with scores of 0–4 were classified as low expression, whereas those with scores of 5–9 were classified as high expression. All images were captured with positive fluorescence microscope (Olympus BX61, Tokyo, Japan).

### WB and antibodies

The cells cultured at 37 °C in 5% CO_2_ were taken out and washed three times with ice-cold PBS (pH 6.8) and then lysed with a lysis buffer (1% sodium dodecyl sulfate (SDS), 10 Mm Tris-Hcl, pH 7.6, 100 mM phenylmethanesulfonyl fluoride) on ice for 10 min. Protein denaturation was performed at 95 °C for 10 min, and then lysates were centrifugated at 12,000 *g* at 4 °C for 10 min followed by collection of the upper clear cell lysates. The protein concentration was measured with Bradford method. Equalized amounts of protein (30–60 µg per line) were loaded, separated by SDS-polyacrylamide gel electrophoresis (PAGE) gels and blotted onto polyvinylidene fluoride (PVDF) membranes. Glyceraldehyde-3-phosphate dehydrogenase (GAPDH) was used as an internal control. Antibodies against the following proteins were used: E-cadherin (1:1000) from Sino Biological Inc.; GAPDH (1:1000), N-cadherin (1:500) from Santa Cruz Biotechnology Inc.; vimentin (1:8000) from Epitomics; PLK4 (1:1000) from Proteintech Group, Inc.; Smad2/3 (1:500) from BD Biosciences, San Diego, CA, USA; Slug (1:1000), Akt (Ser473) (1:1000), p-Akt (Ser473) (1:1000), p-Smad2 (Ser465/467)/Smad3 (Ser423/425) (1:1000), p-Erk1/2 (Thr202/Tyr204) (1:4000), Erk1/2 (1:1000) from Cell Signaling Technology; and goat anti-rabbit and goat anti-mouse secondary antibody from Santa Cruz Biotechnology Inc. (1:4000).

### Real-time PCR

Total RNA from surgically resected fresh NB and relevant normal tissues was extracted using Trizol reagent (Invitrogen, Carlsbad, CA, USA) according to the manufacturer’s instructions. Afterwards, RNA was reverse transcribed into cDNA using a transcriptor First Strand cDNA Synthesis Kit (Invitrogen, Paisley, UK) following the manufacturer’s instructions. Next, quantitative real-time PCR with a Bio-Rad CFX96 system was performed to analyze the amount of cDNA obtained from the above step. The Power SYBR Green Master Mix purchased from Roche was used in the RT-PCR. We detected the expression level of a sample using three separate experiments with triplicate wells per experiment. The amplification reaction repeated 40 cycles. Each cycle contained denaturation at 95 °C for 30 s, annealing for 5 s, and an extension at 60 °C for 30 s. Specific primers for GAPDH (forward, 5′-ACCACAGTCCATGCCATCAC-3′; reverse, 5′-TCCACCACCCTGTTGCTGTA-3′) and PLK4 (forward, 5′-GACACCTCAGACTGAAACCGTAC-3′; reverse, 5′-GTCCTTCTGCAAATCTGGATGGC-3′) were from AuGCT (Beijing, China).

### Cell proliferation assay

Sh-control and sh-PLK4 NB cells were seeded in 96-well plates at a density of 3 × 10^3^ cells per well for initial concentration. Each group of cells set five parallel holes. Then, the cells were incubated with 20 μL MTT (3-(4,5-dimethylthiazol-2-yl)-2, 5-diphenyl-2H-tetrazolium bromide, 5 mg/mL in PBS, Sigma, St. Louis, USA) were added to each well at 37 °C, 5% CO_2_ for 4 h, following which the medium was removed and 150 μL of DMSO (dimethyl sulfoxide, Sigma) was added. The optical density was measured using a micro-plate auto-reader (Bio-Rad). Cell viability was examined at 0, 24, 48, 72, and 96 h.

For colony formation assay, sh-control and sh-PLK4 NB cells were seeded in six-well plates at a density of 0.5 × 10^3^ cells per well. The cells were allowed to grow at 37 °C, 5% CO_2_ for approximately 2 weeks. When they grew to visible colonies, the cells were stained with crystal violet. The colonies were counted and the pictures were taken by digital camera.

### Matrigel invasion assay

Matrigel-coated Transwell inserts (BD Biosciences) were prepared for evaluation of the invasion ability of NB cells before the experiment. The cells were cultured in 24-well plates, and 1 × 10^5^ sh-control and sh-PLK4 NB cells in 200 μL MEM only were plated into the upper chamber with 8-μm pores. Although 600 μL MEM containing 10% FBS was added to the lower chamber. After incubation at 37 °C in 5% CO_2_ for 24 h, noninvaded cells were wiped up with a cotton swab and the invaded cells were fixed with 4% paraformaldehyde and stained. Five randomly selected fields were imaged, and the number of the invaded cells was counted. All images were captured with motorized fluorescent microscopy (Olympus BX61).

### Scratch assay

Sh-control and sh-PLK4 NB cells were plated in six-well plates at a density of 1 × 10^6^ cells/mL and grown overnight. The culture medium used was MEM supplemented with 10% FBS. Cell monolayer was wounded by scratching with 10-μl pipette tip to make an even wound. Next, the cells were washed three times with PBS and cultured with medium containing 0.5% FBS in a humidified incubator maintained at 37 °C, 5% CO_2_. The distance of the wound was recorded and calculated by subtracting the distance between the edges at the appropriate time (3, 6, 9, 12, and 24 h) and the distance measured at 0 h at three random sites. Images were captured at 0 and 24 h with an microscope at ×10 magnification. The data are shown as the mean ± SD.

### Flow cytometry analysis

FITC Annexin V Apoptosis detection kit (BD Pharmingen^TM^) was used to evaluate the apoptosis rate of cells by flow cytometry analysis. The cells grew at a density of 1 × 10^6^ cells per well in 6 cm dish were washed twice with cold PBS and resuspended with 1 × Binding Buffer. In all, 100 μL of the solution was transferred to a 1.5 mL tube, followed with the addition of 5 μL FITC Annexin V and 5 μL 7-AAD. Cells were gently vortexed and incubated at room temperature for 15 min in the dark. Finally, 400 μL 1 × Binding Buffer was added to each tube and made the analysis by flow cytometry within 1 h.

For cell cycle distribution assay, cells were fixed with 95% ethanol at 20 °C overnight. The next day, cells were resuspended in PBS and stained with 500 μL propidium iodide in the dark for at least 30 min.

### Immunofluorescence

Sterile coverslips were placed into 12-well plates, then 4 × 10^4^ cells were plated in each well. The following day, the coverslips were washed with PBS. Cells were fixed with 4% paraformaldehyde for 15 min at room temperature and permeabilized in 0.25% Triton X-100 for 5 min and blocked in 3% BSA at room temperature for 1 h. Cells were afterward incubated with primary antibodies at 4 °C overnight. The next day after rinsing twice with PBS, cells were then stained with an Alexa Fluor 488-conjugated (Invitrogen) secondary antibody at room temperature for 1 h in the dark. Cell nuclei were counterstained with 4’,6-diamidino-2-phenylindole (Roche). Confocal laser scanning microscopy (Olympus FV1000) was used for capturing images.

### pCDH-PLK4 NB cells treated with the LY294002

pCDH-PLK4 NB cells were plated in six-well plates with FBS depleted medium for the first 12 h. Later, LY294002 (Sigma, 10 μmoL, 12 h) was added, and the cells were cultured with medium supplemented with 0.5% FBS at 37 °C in 5% CO_2_. Then, validation of PLK4 overexpression in NB cells was evaluated by WB (shown in Supplementary Figure 2A) and further experiments were made.

### Tumor xenograft transplantation assay

The animal experiments were approved by the Ethics Committee of the Tianjin Medical University Cancer Institute and Hospital. Four-week-old nude mice (Vital River Laboratory Animal Technology Co. Ltd, Beijing, China) were purchased for xenograft animal experiments (*n* = 5 per group). Sh-control/SK-N-BE(2) and Sh-PLK4/SK-N-BE(2) cells were prepared and 2.5 × 10^6^ cells in 100 μL of PBS were injected subcutaneously into the inguens. The tumor volume and weight was monitored twice a week with Vernier caliper and electronic scale. The computational formula of volume was (length × width^2^)/2. Two groups of mice were euthanized when the first one was moribund. To detect whether metastases existed, the lungs, livers, paranephros treated with formalin fixed paraffin embedded were further analyzed with HE staining. The weights and volumes of mice primary tumors were statistically analyzed.

### Statistical methods

SPSS 22.0 was used to evaluate the data. Paired* t*-test was used to assess the expression level of PLK4 in cancer and normal tissues. The relationship between PLK4 expression level and clinicopathological variables were analyzed using *χ*^2^ test. Survival was analyzed using the Kaplan–Meier analysis. OS referred the time interval between diagnosis and the date of death or last follow-up time. PFS was defined as the time from initial diagnosis to the date of progression or death. *P* < 0.05 was defined to be statistically significant.

## Electronic supplementary material

Supplementary Figure 1

Supplementary Figure 2

Supplementary Figure Legend
