# Supplementary figures and images for: Polo-like kinase 4 mediates epithelial–mesenchymal transition in neuroblastoma via PI3K/Akt signaling pathway
Source: Cell Death Dis. 2018 Jan 19;9(2):54. doi: 10.1038/s41419-017-0088-2 (PMC5833556; doi:10.1038/s41419-017-0088-2)

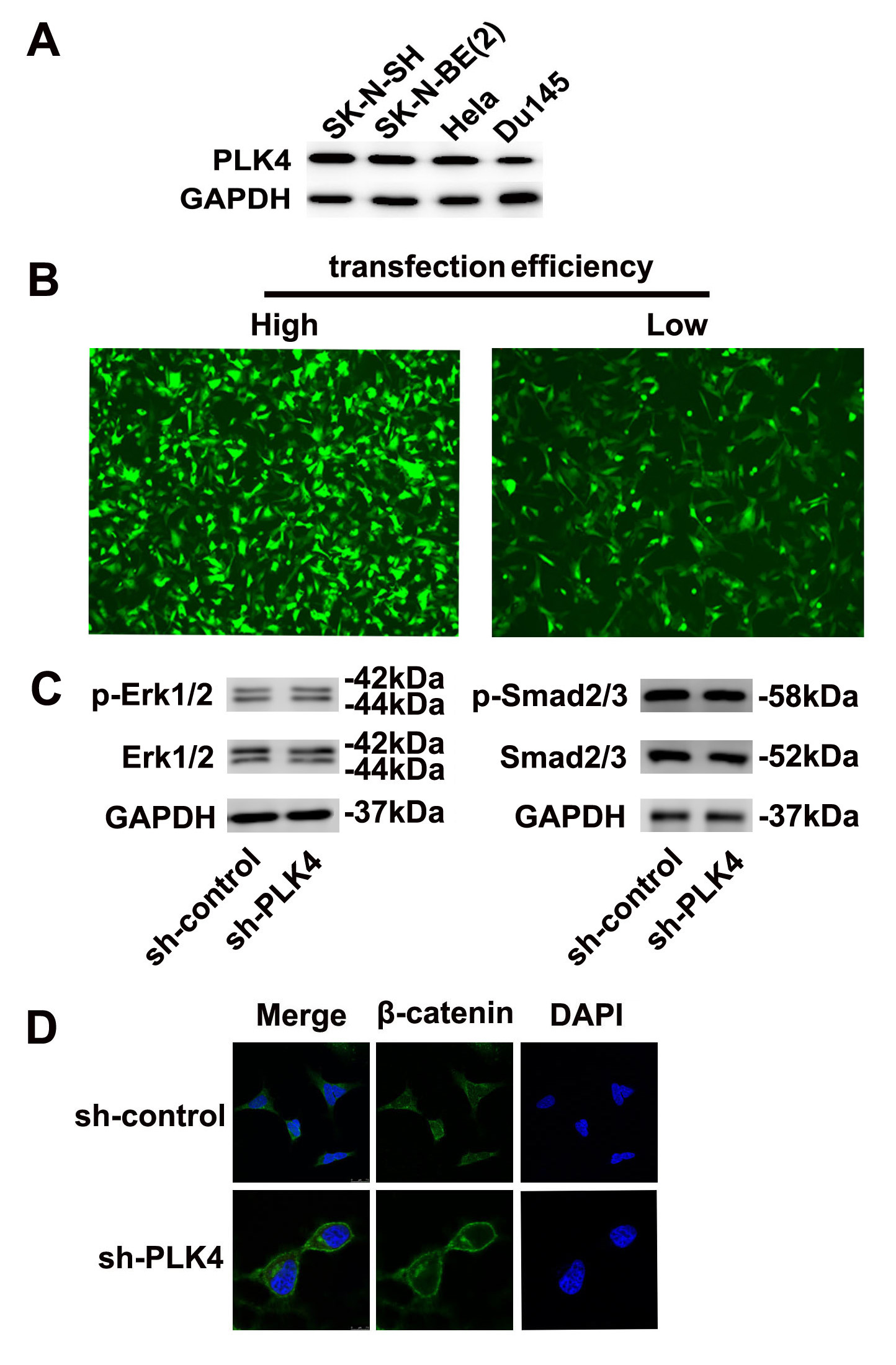

Supplement: Supplementary file 1 — Supplementary Figure 1 [file 41419_2017_88_MOESM1_ESM.jpg]

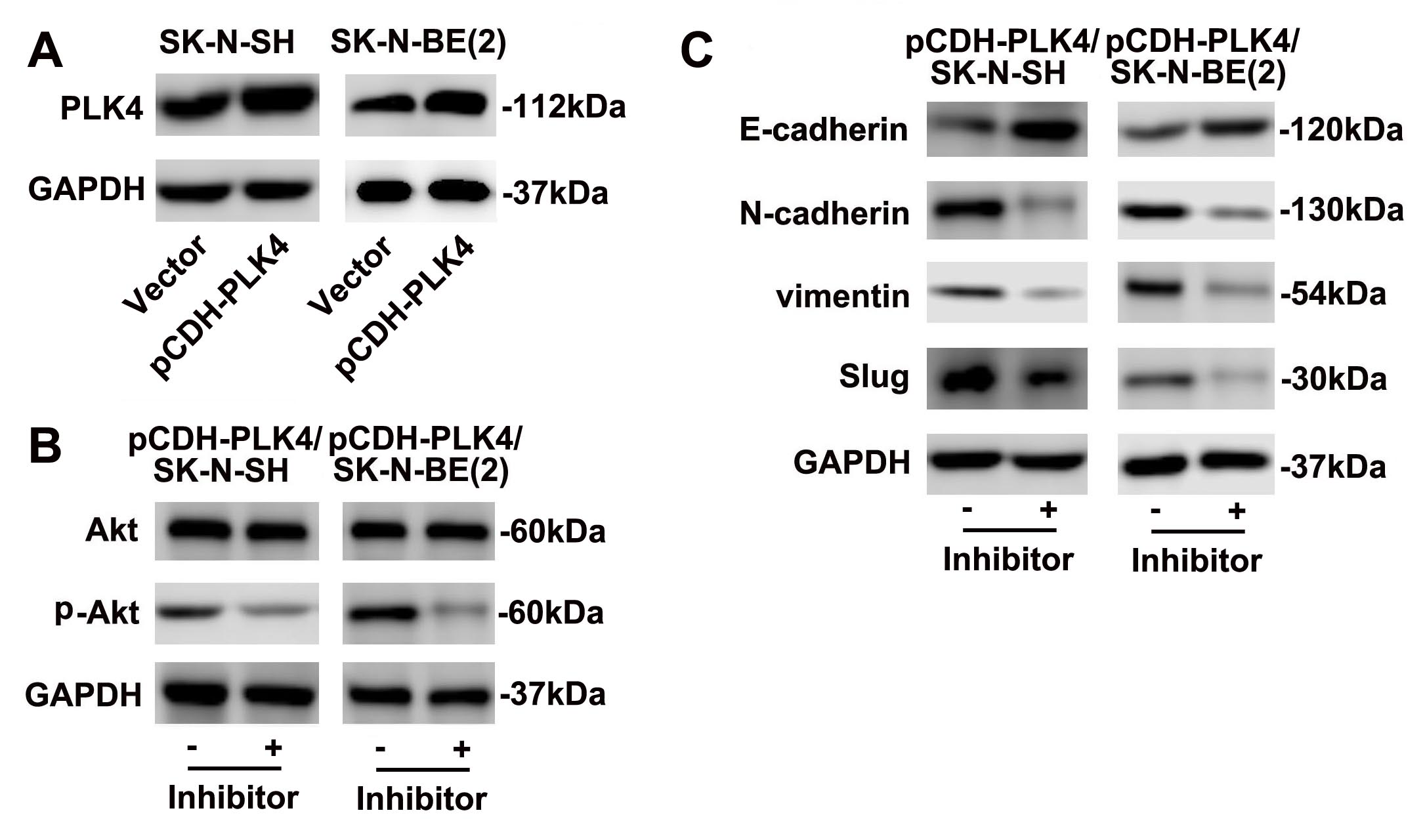

Supplement: Supplementary file 2 — Supplementary Figure 2 [file 41419_2017_88_MOESM2_ESM.jpg]
