# Supplementary material for: Polo-like kinase 4 mediates epithelial–mesenchymal transition in neuroblastoma via PI3K/Akt signaling pathway
Source: Cell Death Dis. 2018 Jan 19;9(2):54. doi: 10.1038/s41419-017-0088-2 (PMC5833556; doi:10.1038/s41419-017-0088-2)
Supplement: Supplementary file 3 — Supplementary Figure Legend [file 41419_2017_88_MOESM3_ESM.docx]

**Supplementary figure Legends**

**Supplementary figure** 1. A. The protein level of PLK4 in different NB cell lines SK-N-SH and SK-N-BE(2) as compared to Hela and Du145 cell lines; B. Comparison of transfection efficiency in SK-N-BE(2) cells with different constructed shRNA plasmids; C. Western blot of Erk1/2 and Smad2/3 in sh-control and sh-PLK4 in SK-N-BE(2) cells; D. Immunofluorescence staining for β-catenin of sh-control and sh-PLK4 in SK-N-BE(2) cells.

**Supplementary figure** 2. A. Validation of PLK4 overexpression after infection in NB cells; Western blot of total Akt, phosphorylated Akt (B) and EMT-associated markers (C) in pCDH-PLK4 NB cells when cultured without and with inhibitor (LY294002 as inhibitor). All experiments were repeated at least three times
